# Image-Based Monitoring of Cracks: Effectiveness Analysis of an Open-Source Machine Learning-Assisted Procedure

**DOI:** 10.3390/jimaging8020022

**Published:** 2022-01-23

**Authors:** Luigi Parente, Eugenia Falvo, Cristina Castagnetti, Francesca Grassi, Francesco Mancini, Paolo Rossi, Alessandro Capra

**Affiliations:** DIEF—Department of Engineering “Enzo Ferrari”, University of Modena and Reggio Emilia, Via Pietro Vivarelli 10, 41125 Modena, Italy; eugenia.falvo@unimore.it (E.F.); cristina.castagnetti@unimore.it (C.C.); francesca.grassi94@unimore.it (F.G.); francesco.mancini@unimore.it (F.M.); paolo.rossi@unimore.it (P.R.); alessandro.capra@unimore.it (A.C.)

**Keywords:** image processing, machine learning, crack, segmentation, monitoring, ImageJ, Ilastik

## Abstract

The proper inspection of a cracks pattern over time is a critical diagnosis step to provide a thorough knowledge of the health state of a structure. When monitoring cracks propagating on a planar surface, adopting a single-image-based approach is a more convenient (costly and logistically) solution compared to subjective operators-based solutions. Machine learning (ML)- based monitoring solutions offer the advantage of automation in crack detection; however, complex and time-consuming training must be carried out. This study presents a simple and automated ML-based crack monitoring approach implemented in open sources software that only requires a single image for training. The effectiveness of the approach is assessed conducting work in controlled and real case study sites. For both sites, the generated outputs are significant in terms of accuracy (~1 mm), repeatability (sub-mm) and precision (sub-pixel). The presented results highlight that the successful detection of cracks is achievable with only a straightforward ML-based training procedure conducted on only a single image of the multi-temporal sequence. Furthermore, the use of an innovative camera kit allowed exploiting automated acquisition and transmission fundamental for Internet of Things (IoTs) for structural health monitoring and to reduce user-based operations and increase safety.

## 1. Introduction

In the design of a construction, it is essential to define a plan of inspections and ordinary maintenance works to guarantee the ‘service life’ (time span in which a structure will maintain the performance levels for which it was designed) [1]. In fact, during its service life a building can be exposed to multiple distress factors that affect it. For example, in reinforced concrete structures, materials are inevitably subject to a progressive degradation due to chemical and physical effects related to the mixture of water, inert and binders, production and installation. Building service life can be affected by external factors also (e.g., load cycles, imposed deformations, temperature variations, vibrations, deformations of the foundation soil, etc.).

According to [2] an accurate evaluation of cracks is a fundamental step for inspection, diagnosis and service life prediction for the safety of concrete structures. The presence of set of cracks is a critical aspect of the structure’s operating behavior, since they are indicators of distress. Cracks can be defined as interruptions of the continuity of a material following stress or deformation states that are not compatible with mechanical characteristics. They can be described and categorized depending on their widths, length and orientation and, to simplify, can be divided as superficial and structural. While superficial cracks do not affect the stability of the structure, monitoring structural crack behavior over time is a critical step to interpret the health state of the construction and for the efficient and timely restoration interventions to heal or rebuild the damaged parts.

Traditionally, visual examination of cracked surfaces is carried by experienced operators who adopt contact surveying tools (e.g., measuring magnifiers, strain gauges, crack rulers, etc.) to measure specific characteristics such as width and length. The recorded characteristics suffer from subjectivity that can critically affect the measurement, particularly when multiple operators carry out the measurements over time. Additionally, for monitoring purposes, it is critical that multi-temporal measurements are repeated on the same spot to correctly evaluate eventual change. These manual monitoring procedures can produce a loss of reliability. A further drawback is that number of measurements along a crack may be restricted to few observations depending on accessibility to the site and on the time and budget available. Recently, innovative crack measuring systems based on fiber optic sensors have been proposed by implanting and bonding the sensors to the monitored surface [3]. Another solution is the adoption of a laser scanner system that allows for generating a high-density and accurate 3D point cloud and achieving satisfactory performance in crack detection [4]. Compared to traditional measurements, such systems can achieve higher accuracy and can overcome the issue of subjective measurements [5]. However, cost and logistical work for the installation and connection of the needed equipment is not advantageous for aged constructions, especially when monitoring a single and localised set of cracks. An image-based crack inspection procedure can offer a solid alternative to the previously described approaches. The adoption of digital images for the inspection of cracks ensures the objectivity of measurements, possibility to achieve sub-mm accuracy [6] and the convenience for quickly recorded and stored characteristics of the entire crack pattern. Additionally, image sequences can be archived, ensuring permanent observations and off-line measurements performable at any time. Furthermore, image acquisition and transferring can be conducted remotely if appropriate protocols are developed [7].

In the case of the deformation of non-planar objects or off-plane movement, adoption of a multi-images-based monitoring system is usually considered [8,9]. However, the use of a single image has proved to be a suitable solution to inspect and measure the temporal evolution of a set of cracks for both two-dimensional (2D) and three-dimensional (3D) applications. For example, the adoption of several targets distributed on both sides of the monitored crack have shown great potential to measure the 2D and 3D displacement of the crack area [10,11]. Indirect measurements deducted from the displacement of targets allow for overcoming some of the most common crack detection-related issues, including poorly segmented cracks (caused by irregular illumination conditions, shading, presence of noise, concrete spall, etc.) and the automation of the processing steps. However, targets with known distances must be installed, and variations are only available along targets limiting the investigation of the observed crack pattern.

Recent advances in hardware and software technology led to a wide growth of image processing-based crack inspection research works over the past few decades. In the literature there are several examples of works focused on the implementation of image-based crack detection approaches for a range of applications [12,13,14,15]. Detailed reviews of works focusing on crack detection through image-processing techniques are reported by [16,17]. When dealing with image processing techniques applied to single images for crack assessment, the majority of works on this topic carry out the recognition, exploiting the crack geometry and the sudden variation of intensity of pixels provoked by the presence of a crack [6,18]. Various image pre-processing algorithms (e.g., multi-scale line filter, median filter, high-pass filter, etc.) are usually applied to single images to remove noise and to enhance the intensity difference in the background (e.g., wall) and foreground (e.g., crack pattern) [16]. However, the presence of features such as uneven finish, voids, stains and shadows can still lead to misinterpretation [19]. Furthermore, reflective surfaces and varied illumination conditions are further challenges to account for when working with image-based monitoring approaches. Therefore, suitable solutions to eliminate all these light variation and noises are required to precisely identify and segment cracks and measure their characteristics [20,21]. After pre-processing, the detection phase can be conducted adopting a range of solutions (e.g., threshold algorithms, geometry-based recognition, mathematical morphology, etc.) depending on the applications and the degree of accuracy desired. Main issues of this phase relate to misinterpretation, loss of the connectivity of detected cracks and noise generation, which can produce false-positive segmentation [22].

Most of the reviewed works develop crack detection approaches based on single images (e.g., single epoch observation). Implementing a standard procedure for multiple crack identification on multi-temporal image sequences is challenging because binarization and following segmentation is dependent on algorithms’ parameters that vary based on specific study site characteristics at the time of image acquisition. Recently, the deployment of machine learning (ML) methods have shown potential to implement automated and real-time systems for varied applications [23,24,25]. The automated detection of crack patterns with ML algorithms has recently been investigated in different scenarios [26,27,28]. Generally, the adoption of ML-based approaches suggests that a huge amount of data in addition to complex and time-consuming training must be carried out to obtain suitable results. However, adopting user-friendly and freely available ML software can represent a suitable solution to the problem.

Besides crack identification, other critical aspects for the development of a complete monitoring solution must be considered (e.g., automated data acquisition and accurate and precise measurement over time). Although the implementation of more complete workflows (including both crack detection and crack analysis) has been proposed by various authors [6,29,30,31,32] further investigation is needed to improve remote and automated capabilities for crack monitoring. In this context, modern monitoring schemes can benefit from recent innovation in transmission and communication networks, which offer the opportunity to develop more complete, reliable and intelligent solutions through the adoption of Internet of Things (IoT) [33]. IoT-based systems, including the latest Internet services and cloud computing technology, can play a key role in implementing the real-time collection, transmission, processing and analysis and visualization of data, which is critical for monitoring both the built environment [33] and territory [34].

The review of the examined studies highlights that despite recent technological advancements in both hardware and software supporting the development of intelligent monitoring systems [35], a complete commercial solution able to provide the tools for innovative predictive maintenance strategies of the built environment is currently not available. Recent research efforts are addressed to the automation of multi-temporal acquisition, processing and analysis, which are critical aspects for modern predictive maintenance strategies and demand minimizing downtime and associated costs. Nowadays, modern open-source image processing and ML software together with advances in data collection and transmission technology offer the tools for the development of a simple near-real time long-term monitoring approach based on a monoscopic system able to detect and analyse cracks with little operator intervention. Thus, the aim of this work is to implement a simple and mostly automated procedure based on open-source software and IoT being able to independently collect and process a sequence of single images of cracked walls for monitoring purposes. Furthermore, the work implements and tests a straightforward ML-based approach where large datasets for pre-training are not required. The effectiveness of the results generated by the proposed approach is assessed using datasets captured in a controlled environment and on a case study that reproduced typical conditions found in buildings with masonry walls.

The novelty of the implemented approach lies in combining an easy-to-implement ML-based approach (for fostering the crack identification automation issue) and IoT capabilities (for achieving multi-temporal and remote data collection) with the overall aim of delivering a reliable cost-effective solution for crack monitoring. The cost-effectiveness is achieved by exploiting open-source algorithms only and by adopting moderately priced devices. Such an affordable solution can be exploited in numerous buildings, thus enabling a diffuse monitoring that is crucial for real estate managers to prioritize maintenance interventions and rationalize the budget. Information on tools used for the entire computational process (from image acquisition to crack analysis) are reported.

This work is carried out in the framework of the InSPiRE project (https://inspire-project.it, accessed on 18 December 2021). The project aims at implementing a predictive diagnostic system for monitoring existing built heritage: materials, systems and components that, under normal operating conditions, reach the end of their useful life. The expected result is an integrated tool for the knowledge of the buildings’ health status and a device to support the predictive maintenance and management of the existing built heritage.

The remainder of the paper is structured as follows: “Section 2” provides a detailed description of the methodology, presenting the architecture of the proposed approach and the two study sites; “Section 3” is dedicated to presenting the main results of this work in terms of the accuracy, precision and repeatability of the proposed approach; the significance of the obtained results and possible extensions of the work are discussed in “Section 4”; finally, in “Section 5”, concluding remarks are summarized.

## 2. Methodology

### 2.1. Architecture of the Approach

**Figure 1:**
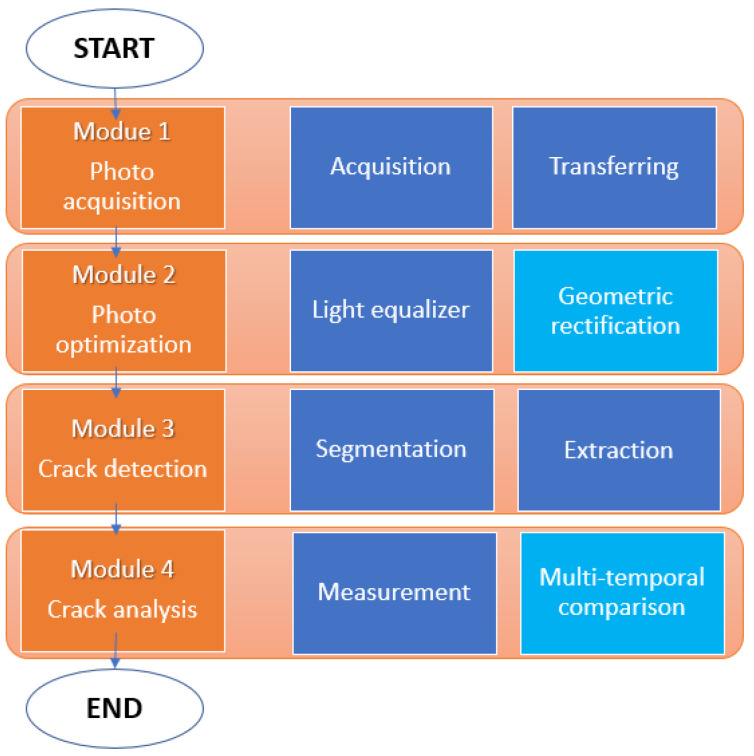
Workflow of the proposed algorithm. Each module includes implemented (blue) and optional actions (light blue).

The proposed algorithm for automatic crack monitoring summarized in Figure 1 consists of four main modules including photo acquisition (PAM), photo optimization (POM), crack detection (CDM) and crack analysis (CAM). Tests of the proposed approach were conducted in controlled conditions (indicated as “laboratory test”) and on a real site (indicated as “on-site test”) representative of a typical scenario of a residential masonry-based plastered wall. The two experimental environments and the four modules are detailed in the following sections.

#### 2.1.1. Photo Acquisition Module (PAM)

**Figure 2:**
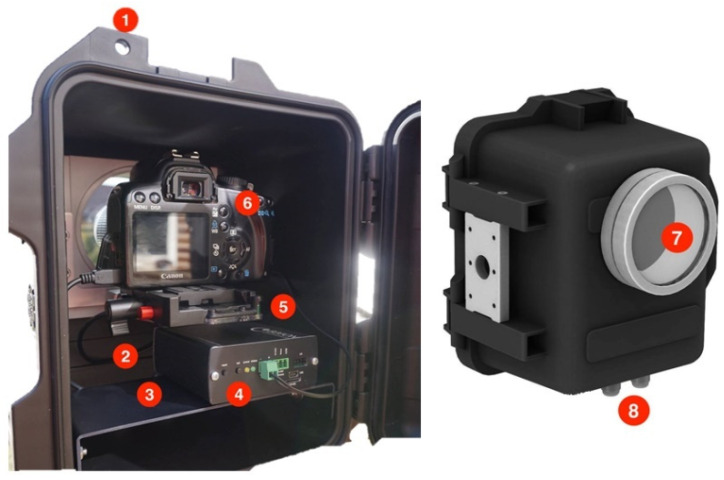
Bixion protective case and components of the acquisition kit including (1) padlock slot; (2) tightening screw; (3) shelf; (4) Bixicon unit; (5) camera slider; (6) DSLR camera; (7) crystal glass flatport ~105 mm UV filter and (8) grommet.

The first step of the proposed procedure is the remote acquisition and transferring of images. The implemented PAM allows for acquiring a sequence of images at predefined intervals of time. The PAM kit includes a single digital single-lens reflex (DSLR) camera, an intervalometer and a protective case (Figure 2). Specifically, a moderately priced Canon 2000D (maximum resolution ~24.1 megapixels and sensor APS-C CMOS) equipped with 18–55 mm lenses was mounted in a protective case. After installation of the acquisition kit in a convenient location (e.g., fixed to a bracket, mounted on a stable tripod, etc.), image capture was performed, automatically adopting a time-lapse controller with remote capabilities. The Bixicon controller [36] was chosen among others for its user-friendly GUI (graphical user interface), unlimited set of image acquisition intervals, remotely controllable settings via WiFi/3G/4G and data transmission capabilities. The 2D input data can be stored in both JPEG and RAW formats on the camera SD and/or in the controller memory. Additionally, acquired images can be transferred using an FTP server or saved on a cloud storage service (e.g., Google Drive, Dropbox, etc.) in just a few seconds. The protective case (dimensions: 23 × 30 × 25 cm; weight: ~5 kg), also provided by Bixion, can be easily adopted for both indoor and outdoor applications. Figure 2 outlines the main components of the case and shows an example of a typical internal configuration adopted for this work.

#### 2.1.2. Photo Optimization Module (POM)

Pre-processing steps are advisable to adjust the acquired image when performing pre-defined feature extraction. The use of a stable camera support (e.g., tripods) and an unchanged artificial illumination can guarantee the stability of camera orientation and radiance of the object. However, in a real environment, the image sequence can be subjected to variations, which can introduce errors in the crack detection and analysis phases. The stability of the camera is especially critical when developing a monitoring system where multi-temporal pixel-to-pixel comparisons are carried out. Small movements of the camera can be corrected adopting a geometric rectification procedure, for example, by implementing algorithms in the processing workflow to correct unwanted camera rotations [37] or by adopting solutions to finely co-registering multi-temporal acquisition [38]. In the POM, camera position is considered stable during the whole acquisition process due to the fixed installation achieved. Thus, only slight variation on the object is accounted for.

The radiometric optimization is implemented using an open-source software, namely ImageJ [39]. This software is user-friendly, contains a manifold of tools and plugins for image processing and allows for creating a macro for batch processing and, thus, automation. The first step for radiometric optimization is to convert the original RGB image to greyscale (8 bit). The application of the algorithms is to subtract the background (“Rolling Ball” plugin) and enhance the contrast, recalculate the pixel values of the original image to uniform brightness and increase smoothness. Parameters used in this study for the radiometric optimization are shown in Appendix A. As a result of the previous optimization steps, the obtained corrective image presents a smother distribution of pixel intensity, while maintaining information related to the presence of cracks.

When working with digital image sequences, calculating a scaling factor is a fundamental step to retrieve metric information. When working with image processing techniques, a common approach adopted to scale images is to use a reference measurement in the image. For example, in the case of measurement of a planar object, a couple of natural or artificial targets with known distance can be considered. It is recognised that the adoption of this scaling option is not appropriate when striving for high accuracy (e.g., sub-mm), but it was used in this work for its quick and easy definition. For high accuracy measurements, it is advised to adopt camera calibration procedures [40]. These allow for accurately estimating camera parameters and reducing systematic errors due to camera distortions. However, even if most of the calibration procedure is highly automated in modern photogrammetric and computer vision software, a skilled operator is needed to set up the calibration field and assess the outputs. Additionally, camera calibration procedures must be repeated when moving the camera to a new site, or if a different camera is adopted. To comply with the goal of developing a simple monitoring procedure camera, calibration was not considered in this study. Furthermore, working with a fixed camera, systematic errors introduced by poor camera calibration are minimised [41].

#### 2.1.3. Crack Detection Module (CDM)

After the optimization phase, images are suitable for the CDM, which classifies the image pixels in classes. A procedure for the semantic segmentation of images via an active learning system [42] is proposed. This module is completely implemented in Ilastik (https://www.ilastik.org, accessed on 18 December 2021). Ilastik is an open-source and user-friendly tool for image classification and segmentation used for multiple applications (e.g., detecting objects, counting spots or cells, time tracking, etc.) on a range of scales. Specifically, the software allows even unexperienced operators to adopt ML-based algorithms to classify image regions in different classes. More detailed information about the software is provided in [43].

Specifically, in the CDM, a single-acquisition machine learning-based training method is proposed. Basically, manual input is only required after the first acquisition (indicated as “reference image”) to train the model. A ‘Pixel Classification’ function is used to perform a binary classification to obtain separating the background (e.g., wall) and the object of interest (e.g., cracks). The operator can choose a set of pixel features (adjustable parameters such as smoothed pixel colour/intensity, edge filters and texture descriptors) and the scales to be considered (for this work, selecting ‘all scales’ for each feature was tested). A mouse interface supports the operator in labelling two classes (namely “crack” and “background”) on the reference image. Thus, such classification allows for assigning labels to each pixel interactively based on predetermined pixel features and on-going user annotations. Based on pixel features and user annotations, the software trains a Random Forest non-linear classifier. The work by [44] is suggested for more technical information about the classifier.

With such an approach, training is only required once on the reference image. The time required to train a robust classifier mainly depends on the user’s ability to correctly label the two classes and the computing resources available. After the classifier has been trained on the reference image, it is ready to be used in batch mode to automatically process all the other images of the dataset. Furthermore, a headless mode can be adopted, which is convenient for running the classifier on a remote machine as a command line. This is advantageous for developing monitoring systems with fully automated capabilities. The results of the image classification and segmentation are then exported as a new image in .tif format to start the next analysis step.

#### 2.1.4. Crack Analysis Module (CAM)

After crack identification, the CAM provides quantitative information. Manual measurements can be implemented along the segmented crack pattern using the measure tool available in ImageJ. However, for this work, a procedure involving the “Ridge Detection” method (plugin available in ImageJ—https://imagej.net/plugins/ridge-detection, accessed on 18 December 2021) was preferred. The potential of this algorithm to automatize the identification and quantification of crack characteristics was already tested by [31,32].

**Figure 3:**
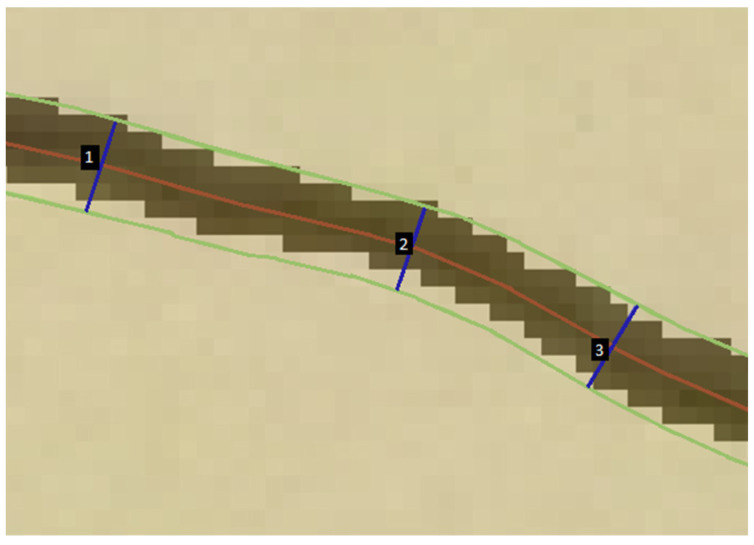
The linear features calculated by the Ridge Detection method include the crack edge lines (green), the maximum line (red) and multiple width segments (blue).

The Ridge Detection method described by [45] is used in image processing and computer vision applications to locate and extract curvilinear structures in digital images. The Ridge Detection method can be used to determine crack geometry, area, length and other properties crucial for maintenance decision making. Specifically, this algorithm estimates the sub-pixel maximum line of the segmented crack and its boundary (edge lines) (Figure 3). The width direction is determined through the normal to the maximum line. Crack width is obtained by calculating the distance between the edge lines measured along the normal line. Width values are calculated with approximately a one-pixel step until there is no crack pixel in the image. Average crack widths of the whole crack pattern are calculated as well. For both test sites, the Ridge Detection default parameters were used to measure crack width and length.

The adopted method estimates metric values for the width and length by multiplying the number of crack pixels by the pixel scaling factor defined in the POM. A multi-temporal comparison step can optionally be added to the CAM. This allows for identifying variation of the monitored cracks over time. However, this further step was not discussed in this work, as the monitored crack patterns of the two test sites were not subjected to change.

### 2.2. Test Sites

Tests were conducted on two different sites to investigate the crack detection and measurement capabilities of the proposed method. Specifically, a laboratory test was performed to assess the accuracy and repeatability provided by the crack monitoring workflow proposed in this work. The same workflow was then optimized for an on-site test, where the analysis focused on the precision of the proposed approach.

#### 2.2.1. Laboratory Test

**Figure 4:**
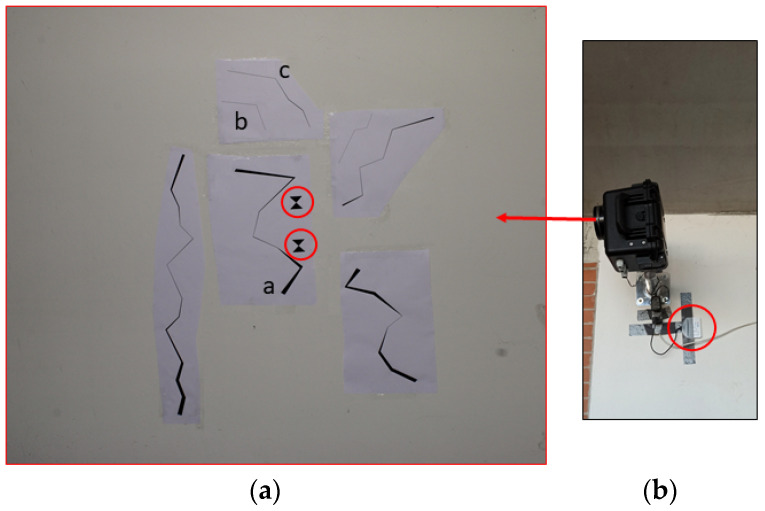
(**a**) The crack pattern generated in AutoCAD for the laboratory test (the red circles indicate the two targets used for scaling purposes, and letters ‘a’, ‘b’ and ‘c’ show the three cracks considered to assess the proposed approach). (**b**) The acquisition kit installed at the laboratory test site (the red circle shows the 4G modem).

The purposes of the laboratory test included an evaluation of the PAM kit performances and an assessment of the proposed method. Specifically, the assessment focused on two main aspects: (1) the ability to detect a range of cracks with various dimensions and orientations and (2) quantifying the measurement accuracy and repeatability. For this reason, a drawing representing a total of seven multi-scale and multi-orientation cracks was realized in AutoCAD (drawing scale of 1:1) (Figure 4a). Cracks were drawn with a wide variation in thickness and length. Specifically, the width varies from a maximum of 1.75 to 0.05 cm. In the drawing, two targets with known size and distance (5 × 5 cm and 17.28 cm, respectively) were added for scaling purposes (Figure 4a).

The CAD drawing was printed on two A0 (118 × 84 cm) white papers. Geometrical characteristics of the printed cracks have been verified using a digital vernier caliper with an uncertainty of ±0.01 mm to ensure that the printing process had not influenced the CAD measures (e.g., targets distance and crack widths). After the positive metricity verification, the crack posters were cropped and attached to an indoor flat wall of an unused indoor room at the Department of Engineering of University of Modena and Reggio Emilia (Figure 4a). Since the light on the laboratory test site was poor, a photography spotlight was used to guarantee homogeneous scene illumination during the whole acquisition process.

A total of four images (namely epoch0_LAB_, epoch1_LAB_, epoch2_LAB_ and epoch3_LAB_) were acquired with a one-minute time span. Epoch0_LAB_ was used as a reference image for the CDM. The four images were captured with the acquisition kit installed to a laboratory wall at 4 m of distance, ensuring the optical camera axis perpendicular to the photographed wall (Figure 4b). A small portable 4G modem was used to ensure a stable internet connection. Acquisition was conducted with the camera in aperture priority (f/11 constant for all images) and autofocus mode, ISO set to 200 and focal length fixed at 27 mm. The above mentioned set up was chosen arbitrarily to simulate acquisition conditions similar to a real case scenario (e.g., monitoring cracks in a small apartment). Based on the camera-object distance and the type of camera and focal length used, the theoretically achievable pixel size on the image projected on to the object is approximately 0.6 mm.

The segmentation outputs of three representative cracks (indicated as crack-a, crack-b and crack-c—see their locations in Figure 4a) were analysed to quantify the measurement accuracy and repeatability. Specifically, the crack geometrical characteristics (length and width) estimated with the proposed approach were compared to the CAD-based measurements.

#### 2.2.2. On-Site Test

The on-site test was carried out to assess the multi-temporal monitoring robustness of the proposed method and to validate it when operating in real conditions (e.g., light variation). The site selected for this experimentation is an indoor masonry wall on the second floor of a four floors residential building located in Bologna (Italy). The building is undergoing restoration works and has been chosen as part of the InSPiRE project as a test site for various monitoring techniques working on a range of scales (e.g., inclinometers, terrestrial laser scanner, satellite radar interferometry, etc.). The investigated portion of the wall is characterized by the presence of multiple cracks with width <0.5 mm and two areas of plaster removed to allow for the characterization of building material.

**Figure 5:**
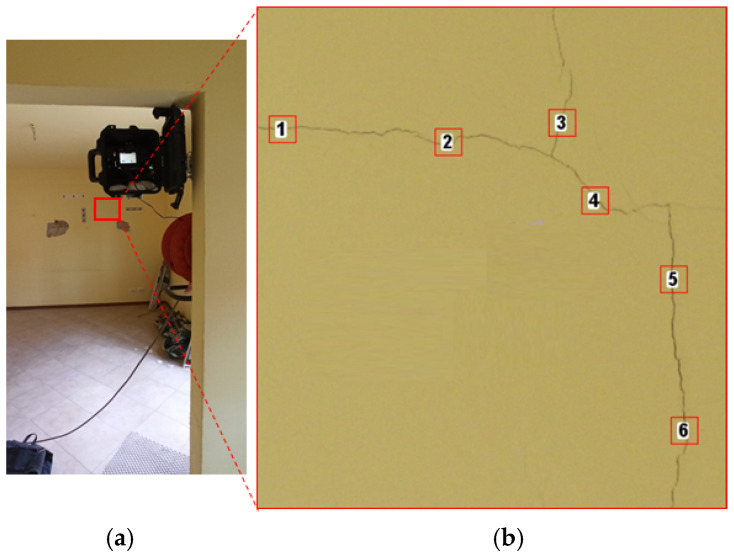
(**a**) The acquisition kit installed indoor for the on-site test. (**b**) Zoom in showing the crack and the locations of the six RoI used to quantify the precision of the proposed approach.

Illumination of the scene is secured by a balcony door that represents only a source of natural daylight for this room. The investigated wall is usually poorly illuminated (as shown in Figure 5a). Illumination of the crack changes depending on the weather and time of the day. The camera was installed on the opposite wall, at approximately 4 m, with the optical axis perpendicular to the photographed scene (Figure 5a). The acquisition of images was performed using the following camera parameters: ISO-200, f/14, auto-focus mode, aperture priority mode and focal length fixed at 37 mm.

The installed acquisition kit and the implemented PAM allowed for independently collecting and transferring images for a period of approximately two months (from 13 May to 17 July 2021). Images were automatically acquired once a week, at 11 am. During the acquisition period, the studied crack pattern was considered stable. This assumption is in accordance with the results provided by contact monitoring sensors installed as part of the InSPiRE project (e.g., network of linear potentiometers and inclinometers).

A sequence of 11 images (namely epoch0_SITE_, epoch1_SITE_, …, epoch10_SITE_) was processed with the approach described in Section 2.1. A cropped area of the photographed site was processed and analysed to favour computational cost. The selected cropped area contains multi-orientation cracks that are not affected by significant variations in terms of crack width.

The results of the on-site test were used to verify the effectiveness of the detection module and the precision of the proposed method. The detection module was first evaluated with a visual assessment of the multi-epoch segmentation and then quantitatively by comparing the automatic segmentation to a ground-truth. The ground-truth was obtained by an operator-based definition of the crack achieved in Ilastik working with the reference image. Specifically, the quantitative analysis was carried out by counting the total number of crack-pixels for all segmented epochs and estimating their percentage against the crack-pixels estimated from the ground truth. To define the precision of the proposed method, multiple RoIs (Region of Interest) with different width sizes were measured over time. Specifically, a total of 6 RoIs (squared region with dimension 50 × 50 pixels) were selected (Figure 5b), and the average width was defined by the Ridge Detection tool for each epoch. The selected RoI were chosen to assess cracks developing in all directions: sub-horizontal (RoI1 and RoI2), oblique (RoI3 and RoI4) and sub-vertical (RoI5 and RoI6).

## 3. Results

### 3.1. Laboratory Test

The developed method (PAM, POM, CDM and CAM) was first applied to the four images acquired during the laboratory test. Training of the classifier using the first image (“reference image”), including the manual labelling and interactive processing, was carried out in approximately 1 h on a laptop Windows 10 Pro with an Intel Core i7-10750H Processor, operating a 2.60 GHz CPU and using 16 GB of RAM. Then remaining images were processed in batch mode, which classified and segmented the crack pattern in only a few seconds.

**Figure 6:**
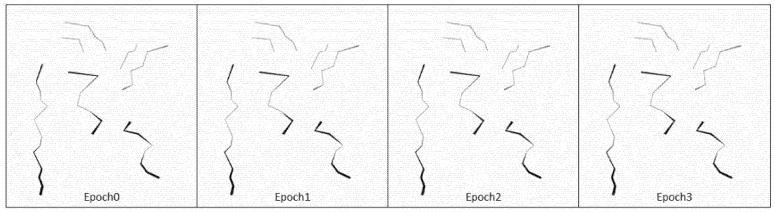
Segmentation outputs generated by the CDM for the laboratory test dataset.

Pixel-level crack segmentation outputs returned by the CDM are shown in Figure 6. Results demonstrate that the proposed approach correctly detected all cracks. Noise effects, false detection or poorly segmented cracks were not observed.

**Figure 7:**
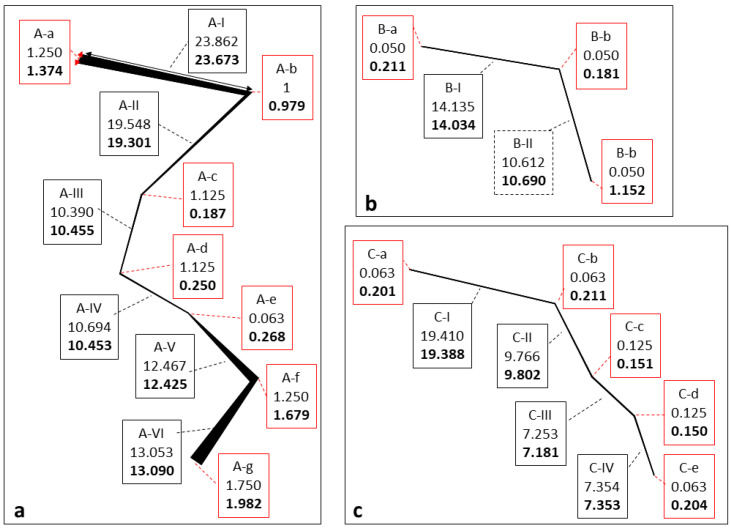
Width (red box) and length (black box) values (in cm) along the three representative cracks (**a**–**c**). Each box includes CrackID, values from the CAD drawing and values estimated with the proposed approach (in bold).

Consequently, each CDM output was inputted in the CAM to automatically define crack measurements. Length and width values calculated with the proposed approach along multiple crack sections (e.g., A-a, A-b, A-I, A-II, etc.) of three representative cracks (crack-a, crack-b and crack-c—Figure 4) were estimated for all epochs. An example of results estimated for epoch0_LAB_ (‘reference image’) are reported in Figure 7. The truth values (estimated from the CAD file) are indicated also. A complete review of length and width measurements and their errors for all epochs is reported in Appendix B. These measurements indicate that, generally, the proposed method can lead to estimating length and width values with sub-mm accuracy. For example, minimum errors of 0.01 mm and 0.20 mm were recorded for length and width, respectively (Appendix B). A few exceptions with measurements differences greater than 2 mm were observed, and in only one case, the proposed method and the ground-truth value differed in all epochs by approximately 4 mm (values referring to a width error—crackID A-f in Appendix B).

To further assess the quality of the laboratory test results, statistics (in terms of mean error and standard deviation) describing the differences between the proposed method and the ground-truth are reported in Table 1 and Table 2. Examining values in Table 1, two main observations can be highlighted: (1) the similarity of differences estimated for all epochs in terms of both, length and width, is significant; and (2) length estimates appear to be more accurate than width ones. These values are in line with the expected theoretical accuracy.

In terms of width, the mean error estimated considering width differences calculated for Crack-a, Crack-b and Crack-c for all epochs is approximately 1.30 mm and decreases to ∼1.10 mm when the crackID A-f is not included in the calculation. As previously noted, the approximately 4mm difference estimated along crackID A-f is repeated for all epochs, demonstrating the presence of a poorly segmented section of the crack. It is interesting to note that such misinterpretation is repeated over time, thus not influencing the repeatability of the method. In order to address this issue, further tests were conducted, demonstrating that improving the training of the classifier can improve the segmentation, decreasing the difference to approximately 2 mm for all epochs.

The test shows that differences values are not influenced by the crack orientation. However, slightly smaller errors are estimated for Crack-b and Crack-c in terms of length and width. The previous cracks have similar orientations and are smaller in size (minimum width is 0.50 and 0.63 mm, respectively) when compared to Crack-a (width ranges between 0.63 and 12.50 mm).

In Table 2, average standard deviations calculated from values of length and width of corresponding sections (from epoch0_LAB_ to epoch3_LAB_) demonstrate an acceptable level of repeatability of the measurement (sub-mm). Maximum standard deviations calculated for length and width values correspond to 0.60 mm and 0.50 mm, respectively, revealing again the high level of repeatability of the proposed method.

### 3.2. On-Site Test

**Figure 8:**
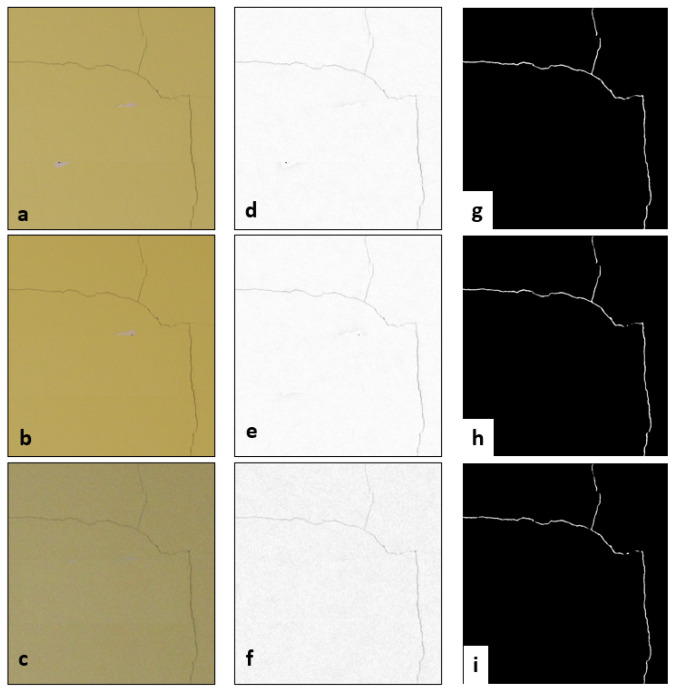
An example of not uniform illumination conditions on multi-temporal sequence of images acquired with natural daylight (**a**–**c**) and the respective optimized images (**d**–**f**) and segmentation outputs (**g**–**i**).

Figure 8a–c shows three representative examples of acquired images with illumination variations selected from the on-site dataset. The POM produced a new set of images where uneven illumination and emphasized crack pixels are observable (Figure 8d–f). The crack detection results plotted in Figure 8g–i are satisfactory considering that most of the crack was correctly identified, and noise or other features were not detected. As illustrated in Table 3, for most epochs, a good level of completeness of automatically detected cracks is achieved (above 90%). On the other hand, with respect to epochs 7_SITE_, 8_SITE_ and 9_SITE_, the performance of the automatic detection was lower. In fact, only approximately between 64 and 69% of the crack pixels were correctly detected when compared to the ground-truth.

Regarding the robustness of the method in estimating crack width over time, results of the six RoI-based analyses are reported in Table 4. Most of the observations (RoI1, RoI3 and RoI4) highlight that the tested approach is capable of estimating similar width values differing by approximately 1 pixel. For RoI5 and RoI6 an even better precision of the method is recorded (sub-pixel precision). However, the RoI-based test also demonstrated that significant (e.g., several pixels) apparent changes in crack width estimation may be recorded (as shown for RoI2 in epochs 7_SITE_, 8_SITE_ and 9_SITE_). Such differences may be erroneously interpreted as crack variations. Indeed, they are a consequence of the poor segmentation outputs obtained for the corresponding RoI.

The technical cause (e.g., use of different acquisition camera parameters) producing an evident difference in crack segmentation for RoI2 in epochs 7_SITE_, 8_SITE_ and 9_SITE_ is not clear. It is suggested that it is due solely to a limit of the classification algorithm used in Ilastik and is not dependent on the operator labelling or on the use of certain camera parameters. The RoI-based analysis also highlights that the proposed procedure is more precise when the analysed crack is sub-vertical (RoI5 and RoI6). At the same time, it can be observed that the previously described effect of crack misinterpretation in epoch 7_SITE_, 8_SITE_ and 9_SITE_, producing minor differences also for RoI1, 3 and 4, did not influence the analysis of sub-vertical cracks (RoI5 and RoI6).

In summary, the on-site test results suggested that (1) the proposed method can produce robust segmentation, although a poorly reconstructed area can still affect the outcome; (2) a high precision of crack detection can be achieved over time with segmentation outputs highlighting sub-pixel repeatability; (3) the geometry of the detected feature can influence the quality of the detection; and (4) the generation of the unaccountable false-negative segmentation of pixels can influence the multi-temporal analysis.

## 4. Discussion

The need to develop an image-based crack monitoring system that easy to use and has little user intervention has led to the implementation of four operational modules and their assessment under controlled conditions and at a real site. The main peculiar aspects characterizing the proposed system include (1) the potential for continuous monitoring providing timely detection and analysis of the status of the feature of interest (e.g., set of cracks); (2) the simplicity and inexpensiveness as the whole classification with an analysis workflow being carried out with freely available machine learning and image processing software that requires little user intervention; and (3) remote and IoT abilities so it can be operated remotely (increasing safety) via a smart image acquisition kit (note any digital camera can be potentially used) and a remote computer (or an on-site small single-board computer—for example, a Raspberry Pi) to run the proposed algorithm (via IoT protocols) and to store a database containing the measurement history of the monitored site. These aspects are in line with the current need to implementing smart structural health monitoring schemes [33].

Furthermore, the literature review demonstrated that developing ad-hoc deep convolutional neural networks (DCNN) for crack detection has been largely adopted in recent years [13,14,28,46]. The presented CDM offers an innovative single-acquisition, machine learning-based training solution that avoids the need to use extensive dataset containing images of wall cracks captured on-site [46] or pre-captured and pre-classified [15,47].

The proposed method proved its efficiency in generating noise-free segmentation, avoiding the need for manual operations and further post-processing that can affect the quality of the crack detection [22,30]. The error in automated crack detection and measurements is generally caused by a range of different sources, including the illumination of the environment [31] and the presence on the investigated structural surface of other discontinuities (e.g., stains, scratching, etc.) [19]. The adoption of a uniform illumination on the site (see the ‘laboratory test’) has certainly favoured the results in terms of segmentation. When dealing with a set of images acquired with varied illumination conditions, the adoption of radiometric corrections used for the on-site test has been demonstrated to be a suitable solution. This is in accordance with other crack detection methods that have opted for similar corrections [2,22]. The adoption of the proposed CDM allows the user to filter out all unwanted features present on the monitored surface. This has the potential to significantly reduce a common issue related to the inclusion of no-crack related features [16,17]. This is advantageous when working in challenging conditions (e.g., structural surfaces with various sources of noises), and further investigation must be conducted to assess the real potential in such contexts.

The overall quality of the crack detection method proposed in this study proved to be satisfactory, allowing for correctly detecting most of the crack patterns examined. The laboratory test shows that misinterpretation can be avoided when the classifier is properly trained. In fact, despite their smaller size, crack-b and crack-c produced slightly better results (smaller errors—Table 1) because for these cracks the operator conducted a better labelling producing an improvement in the segmented result. However, this case is not confirmed with the on-site test, where even a well-trained classifier can still produce an erroneous classification of pixels leading mainly to false negative outputs. As a result, for some epochs of the on-site test, the proposed approach has a tendency to fail crack detection. Thus, portions of the cracks are classified as non-crack, resulting in loss of the connectivity of detected cracks. This is a common issue with image processing and machine learning-based detection algorithms [26] that is usually faced with the adoption of morphological operators [8,9]. Such solutions were not considered for the current study but must be implemented for a future improvement of the proposed approach to increase the reliability of the monitoring system.

An alternative to morphological operators may be to conduct further experiments investigating the use of other ML-based approaches recently adopted for crack detection applications [27]. Another solution is the adoption of a confidence threshold based on the percentage of crack pixels correctly detected. For example, to decide whether the processing of an image generated an acceptable segmentation, the comparison with a confidence threshold (e.g., percentage of crack pixels obtained from the reference image) can avoid considering epochs with poorly segmented cracks that can be interpreted as a change of the crack (e.g., false negative).

To obtain accurate metric crack analysis, the adoption of proper camera calibration procedures, which require an experienced operator and that must be repeated over time, is advisable. However, in accordance with results published by [6], accurate results in crack detection can be still achieved even when camera calibration procedures are not implemented. In fact, the approach proposed in this study has demonstrated that an accuracy of ±1.10 mm and ±0.50 mm (width and length mean error, respectively) can be achieved with an uncalibrated low-cost digital camera mounting a zoom lens. The achieved accuracy proved to be relatively more accurate when compared to other image-based crack monitoring procedures [12,29]. For crack monitoring applications where a better accuracy is required, the proposed approach is expected to notably improve by decreasing the camera-object distance and by using a digital camera with a higher resolution or mounting a macro lens.

The repeatability of the approach is suitable for most monitoring applications. The laboratory and on-site tests suggest that sub-mm (>0.2 mm) and sub-pixel precision can be achieved, respectively. However, the previously described false-negative outputs require further investigation to ensure that the estimated precision outputs are constant along the whole crack pattern.

The proposed approach based on the combination of ImageJ, Ilastik and the Bixion acquisition kit can greatly reduce the crack inspection time and lead to greater accuracy for long-term monitoring when compared to traditional and subjective approaches. It is recognised that the presented single-acquisition machine learning-based training is better suited for applications where the set of crack site is localised and well defined. For different scenarios (e.g., multiple set of cracks with undefined locations) other solutions may be more productive and convenient (timely and costly). In these cases, the inspection and monitoring of structural damages such as cracks can benefit from the usage of Unmanned Aerial Vehicles (UAVs) [14,28,48].

## 5. Conclusions

This research has demonstrated the effectiveness of an image-based approach for crack monitoring that requires little user intervention and experience. Specifically, the implemented image-based procedure proposes a solution to automate remote crack inspection over time using a single digital camera. The adoption of a simple segmentation procedure (single training using a single image) has been proposed, showing great potential to discriminate cracks, even from differently illuminated backgrounds.

Two study sites have been considered to test the potential of the approach, including a laboratory site with controlled conditions (e.g., stable illumination) and an indoor wall of a residential building. A CAD drawing was used, highlighting the potential of the proposed approach in terms of both the detection and accuracy of the measurement. Multi-temporal acquisition was conducted on both test sites to test the repeatability and precision of the approach, proving that results in the scale of sub-mm and sub-pixel can be achieved. The remote and IoT potential of the approach have been discussed as well, demonstrating that automated crack inspection is guaranteed over time by using cost-effective tools.

In summary, this study suggests that the presented approach can offer an objective and efficient alternative to the conventional human-based method for the inspection of cracks and may represent a simplified automated detection solution when compared to more complex DCNNs. Additionally, the approach is scalable and can be easily replicate, as only open-source algorithms and moderately priced devices were adopted.

This investigation showed potential directions for future improvements. Solving the false-negative detection problem is a critical step to improve the monitoring capabilities of the proposed system. Thus, future work must focus on the discussed improvements of the CDM and implementation of further-improved automation of the whole task (e.g., scaling can be automatized with automated target detection). The identification abilities of the proposed approach when working on different sites with more complex backgrounds (for example with cracks superimposed on other textures such as coloured tiles or with the presence of substantial noise) needs investigation to better define the scalability and adaptability of the approach. Finally, further research must be conducted to exploit the potential of integrating current technological advancements (in terms of UAVs and open-source software such as ImageJ and Ilastik) to conduct automated inspections of the built environment. The adoption of modern commercial UAVs with 4G internet connectivity, AI-assisted navigation, high resolution built-in camera and customizable and modular software will play a key role in structural health monitoring schemes by reducing the risk, time and cost of the whole task, essentials aspects with regard to the development of smart cities.

## Figures and Tables

**Table 1:** Summarization of mean error values (cm) computed from all epochs of the laboratory test results.

Mean Error	Epoch0_LAB_	Epoch1_LAB_	Epoch2_LAB_	Epoch3_LAB_
Width (cm)	0.135	0.130	0.126	0.128
Length (cm)	−0.044	−0.052	−0.056	−0.062

**Table 2:** A summary of standard deviation values computed from all epochs of the laboratory test results. Note: the shown values are averages of standard deviation values calculated for the three representative cracks (a, b and c).

St. Deviation	Crack-a	Crack-b	Crack-c
Width (cm)	0.019	0.007	0011
Length (cm)	0.025	0.026	0.012

**Table 3:** Comparison of completeness of automatic crack detection for all epochs of the on-site test.

Epoch	Total Crack-Pixel	%
Ground-truth	6972	100
1	6693	96
2	6321	90.7
3	6673	95.7
4	6786	97.3
5	5906	84.7
6	6365	91.3
7	4796	68.8
8	4528	64.9
9	4503	64.6
10	5896	84.6

**Table 4:** Average crack width (in pixels) estimated for each RoI from epoch0_SITE_ to epoch10_SITE_.

Epoch	RoI1	RoI2	RoI3	RoI4	RoI5	RoI6
0	5.2	5.3	5.5	5.8	5.0	5.7
1	5.4	5.1	5.3	5.9	5.0	5.7
2	4.8	5.0	5.0	5.4	5.2	5.3
3	5.4	5.4	5.5	6.0	5.2	5.6
4	5.3	5.4	5.5	6.1	5.1	5.8
5	5.2	4.6	4.8	5.7	5.6	5.4
6	5.2	4.8	4.8	5.4	5.7	5.8
7	4.4	3.0	4.1	5.3	5.7	5.7
8	4.4	3.8	4.2	4.8	5.6	5.4
9	4.4	2.8	4.4	5.2	5.5	5.5
10	5.0	4.6	4.7	5.8	5.5	5.6

## Data Availability

The data is available upon reasonable request to the corresponding author (luigi.parente@unimore.it).

## Appendix A. Radiometric Optimization—ImageJ Macro

run(“8-bit”);

run(“Enhance Contrast…”, “saturated = 0.3 normalize”);

run(“Enhance Contrast…”);

run(“Subtract Background…”, “rolling = 50 light”);

## Appendix B. Additional Table Including All Results Obtained in the Laboratory Test

	**Length (cm)**				**Width (cm)**			
	**Crack ID**	**Proposed Method**	**Ground-Truth**	**Error**	**Crack ID**	**Proposed Method**	**Ground-Truth**	**Error**
Epoch0_LAB_	A-I	23.673	23.862	−0.189	A-a	1.374	1.250	0.124
	A-II	19.301	19.548	−0.247	A-b	0.979	1.000	−0.021
	A-III	10.455	10.390	0.065	A-c	0.187	0.125	0.062
	A-IV	10.453	10.694	−0.241	A-d	0.250	0.125	0.125
	A-V	12.425	12.467	−0.042	A-e	0.268	0.063	0.206
	A-VI	13.090	13.053	0.037	A-f	1.679	1.250	0.429
					A-g	1.982	1.750	0.232
	B-I	14.034	14.037	−0.003	B-a	0.211	0.050	0.161
	B-II	10.690	10.612	0.078	B-b	0.181	0.050	0.131
					B-c	0.152	0.050	0.102
	C-I	19.388	19.410	−0.022	C-a	0.201	0.063	0.139
	C-II	9.766	9.802	−0.036	C-b	0.211	0.063	0.149
	C-III	7.253	7.181	0.072	C-c	0.151	0.125	0.026
	C-IV	7.354	7.353	0.001	C-d	0.150	0.125	0.025
					C-e	0.204	0.063	0.142
Epoch1_LAB_	A-I	23.591	23.862	−0.271	A-a	1.374	1.250	0.124
	A-II	19.299	19.548	−0.249	A-b	0.979	1.000	−0.021
	A-III	10.443	10.390	0.053	A-c	0.232	0.125	0.107
	A-IV	10.465	10.694	−0.229	A-d	0.185	0.125	0.060
	A-V	12.447	12.467	−0.020	A-e	0.229	0.063	0.167
	A-VI	13.022	13.053	−0.031	A-f	1.679	1.250	0.429
					A-g	1.988	1.750	0.238
	B-I	14.034	14.037	−0.003	B-a	0.211	0.050	0.161
	B-II	10.637	10.612	0.025	B-b	0.184	0.050	0.134
					B-c	0.152	0.050	0.102
	C-I	19.388	19.410	−0.022	C-a	0.200	0.063	0.138
	C-II	9.766	9.802	−0.036	C-b	0.185	0.063	0.123
	C-III	7.253	7.181	0.072	C-c	0.189	0.125	0.064
	C-IV	7.435	7.353	0.082	C-d	0.116	0.125	−0.009
					C-e	0.201	0.063	0.139
Epoch2_LAB_	A-I	23.536	23.862	−0.326	A-a	1.304	1.250	0.054
	A-II	19.270	19.548	−0.278	A-b	0.979	1.000	−0.021
	A-III	10.430	10.390	0.040	A-c	0.228	0.125	0.103
	A-IV	10.465	10.694	−0.229	A-d	0.147	0.125	0.022
	A-V	12.447	12.467	−0.020	A-e	0.279	0.063	0.217
	A-VI	13.022	13.053	−0.031	A-f	1.679	1.250	0.429
					A-g	1.978	1.750	0.228
	B-I	14.034	14.037	−0.003	B-a	0.211	0.050	0.161
	B-II	10.690	10.612	0.078	B-b	0.188	0.050	0.138
					B-c	0.156	0.050	0.106
	C-I	19.388	19.410	−0.022	C-a	0.201	0.063	0.139
	C-II	9.766	9.802	−0.036	C-b	0.165	0.063	0.103
	C-III	7.253	7.181	0.072	C-c	0.166	0.125	0.041
	C-IV	7.435	7.353	0.082	C-d	0.154	0.125	0.029
					C-e	0.202	0.063	0.140
Epoch3_LAB_	A-I	23.536	23.862	−0.326	A-a	1.302	1.250	0.052
	A-II	19.270	19.548	−0.278	A-b	0.979	1.000	−0.021
	A-III	10.415	10.390	0.025	A-c	0.228	0.125	0.103
	A-IV	10.465	10.694	−0.229	A-d	0.219	0.125	0.094
	A-V	12.447	12.467	−0.020	A-e	0.279	0.063	0.217
	A-VI	13.022	13.053	−0.031	A-f	1.679	1.250	0.429
					A-g	1.978	1.750	0.228
	B-I	13.982	14.037	−0.055	B-a	0.211	0.050	0.161
	B-II	10.691	10.612	0.079	B-b	0.149	0.050	0.099
					B-c	0.153	0.050	0.103
	C-I	19.388	19.410	−0.022	C-a	0.201	0.063	0.139
	C-II	9.766	9.802	−0.036	C-b	0.165	0.063	0.103
	C-III	7.253	7.181	0.072	C-c	0.166	0.125	0.041
	C-IV	7.435	7.353	0.082	C-d	0.152	0.125	0.027
					C-e	0.204	0.063	0.142

## References

[1] MIT (2018). Italian Ministry of Infrastructure and Transport. Aggiornamento delle «Norme tecniche per le costruzioni». Gazzetta Ufficiale.

[2] Shan B., Zheng S., Ou J. (2016). A stereovision-based crack width detection approach for concrete surface assessment. KSCE J. Civ. Eng..

[3] Barrias A., Casas J.R., Villalba S. (2018). Embedded distributed optical fiber sensors in reinforced concrete structures—A case study. Sensors.

[4] Feng H., Li W., Luo Z., Chen Y., Fatholahi S.N., Cheng M., Wang C., Junior J.M., Li J. (2021). GCN-Based Pavement Crack Detection using mobile LiDAR point clouds. IEEE Trans. Intell. Transp. Syst..

[5] Bellagamba I., Caponero M., Mongelli M. (2019). Using fiber-optic sensors and 3D photogrammetric reconstruction for crack pattern monitoring of masonry structures at the Aurelian Walls in Rome, Italy. WIT Trans. Built Environ..

[6] Barazzetti L., Scaioni M. (2009). Crack measurement: Development, testing and applications of an automatic image-based algorithm. ISPRS J. Photogramm. Remote Sens..

[7] Chang T., Lee L. (2018). Automatic monitoring system based on IoT and vision technology. Preprints.

[8] Jahanshahi M.R., Masri S.F., Padgett C.W., Sukhatme G.S. (2013). An innovative methodology for detection and quantification of cracks through incorporation of depth perception. Mach. Vis. Appl..

[9] Galantucci R.A., Fatiguso F. (2019). Advanced damage detection techniques in historical buildings using digital photogrammetry and 3D surface anlysis. J. Cult. Herit..

[10] Barazzetti L., Scaioni M. (2010). Development and implementation of image-based algorithms for measurement of deformations in material testing. Sensors.

[11] Nishiyama S., Minakata N., Kikuchi T., Yano T. (2015). Improved digital photogrammetry technique for crack monitoring. Adv. Eng. Inform..

[12] Zhang W., Zhang Z., Qi D., Liu Y. (2014). Automatic crack detection and classification method for subway tunnel safety monitoring. Sensors.

[13] Sizyakin R., Cornelis B., Meeus L., Martens M., Voronin V., Pižurica A. (2018). A deep learning approach to crack detection in panel paintings. Comput. Sci..

[14] Vazquez-Nicolas J.M., Zamora E., Gonzalez-Hernandez I., Lozano R., Sossa H. Towards automatic inspection: Crack recognition based on Quadrotor UAV-taken images. Proceedings of the International Conference on Unmanned Aircraft Systems.

[15] Özgenel F., Gönenç Sorguç A. Performance comparison of pretrained convolutional neural networks on crack detection in buildings. Proceedings of the ISARC 2018 35th International Symposium on Automation and Robotics in Construction.

[16] Mohan A., Poobal S. (2018). Crack detection using image processing: A critical review and analysis. Alexandria Eng. J..

[17] Agnes Shifani S., Thulasiram P., Narendran K., Sanjay D.R. A study of methods using image processing technique in crack detection. Proceedings of the 2nd International Conference on Innovative Mechanisms for Industry Applications.

[18] Yamaguchi T., Nakamura S., Saegusa R., Hashimoto S. (2008). Image-based crack detection for real concrete surfaces. IEEJ Trans. Electr. Electron. Eng..

[19] Valença J., Dias-Da-Costa D., Júlio E., Araújo H., Costa H. (2013). Automatic crack monitoring using photogrammetry and image processing. Meas. J. Int. Meas. Confed..

[20] Schmugge S.J., Rice L., Lindberg J., Grizziy R., Joffey C., Shin M.C. Crack segmentation by leveraging multiple frames of varying illumination. Proceedings of the IEEE Winter Conference on Applications of Computer Visions.

[21] Öztürk Ş., Akdemir B. (2018). Fuzzy logic-based segmentation of manufacturing defects on reflective surfaces. Neural Comput. Appl..

[22] Fujita Y., Hamamoto Y. (2011). A robust automatic crack detection method from noisy concrete surfaces. Mach. Vis. Appl..

[23] Öztürk Ş., Akdemir B. (2018). Real-time product quality control system using optimized Gabor filter bank. Int. J. Adv. Manuf. Technol..

[24] Shahbazi Z., Byun Y.C. (2021). A procedure for tracing supply chains for perishable food based on blockchain, machine learning and fuzzy logic. Electronics.

[25] Weidner L., Walton G., Kromer R. (2019). Classification methods for point clouds in rock slope monitoring: A novel machine learning approach and comparative analysis. Eng. Geol..

[26] Kim J.J., Kim A.R., Lee S.W. (2020). Artificial neural network-based automated crack detection and analysis for the inspection of concrete structures. Appl. Sci..

[27] Hsieh Y.-A., Tsai Y.J. (2020). Machine learning for crack detection: Review and model performance comparison. J. Comput. Civ. Eng..

[28] Ko P., Prieto S.A., García de Soto B. ABECIS: An automated building exterior crack inspection system using UAVs, open-source deep learning and photogrammetry. Proceedings of the 38th International Symposium on Automation and Robotics in Construction.

[29] Lins R.G., Givigi S.N. (2016). Automatic crack detection and measurement based on image analysis. IEEE Trans. Instrum. Meas..

[30] Albareda-Valls A., Herrera A.B., Mestre J.L.Z., Zaribaf S.S. (2018). Image post-processing method for quantification of cracking in RC precast beams under bending. Buildings.

[31] Mahfuzur Rahman M., Saifullah I., Kumar Ghosh S. (2019). Detection and measurements of cracks in axially loaded tension RC members by image processing technique. Am. J. Civ. Eng. Archit..

[32] Meyer C.S., Bonyi E., Drake K., Obafemi-Babatunde T., Daodu A., Ajifa D., Bigio A., Taylor J., Haque B.Z., O’Brien D.J. (2021). Automated detection and quantification of transverse cracks on woven composites. J. Reinf. Plast. Compos..

[33] Scuro C., Sciammarella P.F., Lamonaca F., Olivito R.S., Carni D.L. (2018). IoT for structural health monitoring. IEEE Instrum. Meas. Mag..

[34] Saponaro M., Capolupo A., Caporusso G., Reina A., Fratino U., Tarantino E. Exploring UAV and cloud platform potentialities for detecting geomorphological changes in coastal environment. Proceedings of the Protection and Restoration of the Environment.

[35] Sony S., Laventure S., Sadhu A. (2019). A literature review of next-generation smart sensing technology in structural health monitoring. Struct. Control Health Monit..

[36] (2020). Bixion BixiCon III—User Guide. https://www.bixion.com/BixiCon-manual.pdf.

[37] Roncella R., Forlani G., Fornari M., Diotri F. (2014). Landslide monitoring by fixed-base terrestrial stereo-photogrammetry. ISPRS Ann. Photogramm. Remote Sens. Spat. Inf. Sci..

[38] Parente L., Chandler J.H., Dixon N. (2021). Automated registration of SfM-MVS multitemporal datasets using terrestrial and oblique aerial images. Photogramm. Rec..

[39] Schneider C.A., Rasband W.S., Eliceiri K.W. (2012). NIH Image to ImageJ: 25 years of image analysis. Nat. Methods.

[40] Luhmann T., Fraser C., Maas H.G. (2016). Sensor modelling and camera calibration for close-range photogrammetry. ISPRS J. Photogramm. Remote Sens..

[41] Parente L., Chandler J.H., Dixon N. (2019). Optimising the quality of an SfM-MVS slope monitoring system using fixed cameras. Photogramm. Rec..

[42] Kan A. (2017). Machine learning applications in cell image analysis. Immunol. Cell Biol..

[43] Berg S., Kutra D., Kroeger T., Straehle C.N., Kausler B.X., Haubold C., Schiegg M., Ales J., Beier T., Rudy M. (2019). Ilastik: Interactive machine learning for (bio)image analysis. Nat. Methods.

[44] Geurts P., Irrthum A., Wehenkel L. (2009). Supervised learning with decision tree-based methods in computational and systems biology. Mol. Biosyst..

[45] Steger C. (1998). An Unbiased Detector of Curvilinear Structures. IEEE Trans. Pattern Anal. Mach. Intell..

[46] Li S., Zhao X. (2019). Image-based concrete crack detection using convolutional neural network and exhaustive search technique. Adv. Civ. Eng..

[47] Dorafshan S., Thomas R.J., Maguire M. (2018). SDNET2018: An annotated image dataset for non-contact concrete crack detection using deep convolutional neural networks. Data Brief.

[48] Germanese D., Leone G.R., Moroni D., Pascali M.A., Tampucci M. (2018). Long-term monitoring of crack patterns in historic structures using UAVs and planar markers: A preliminary study. J. Imaging.

